# Protective effects of troxerutin on maternal high-fat diet-induced impairments of spatial memory and apelin in the male offspring

**DOI:** 10.22038/IJBMS.2018.28170.6901

**Published:** 2018-07

**Authors:** Roghayeh Diba, Gisou Mohaddes, Fariba Mirzaie Bavil, Fereshteh Farajdokht, Parvin Bayandor, Maryam Hosseindoost, Keivan Mehri, Zohreh Zavvari Oskuye, Shirin Babri

**Affiliations:** 1 Drug Applied Research Center, Tabriz University of Medical Sciences, Tabriz, Iran; 2 Neurosciences Research Center, Tabriz University of Medical Sciences, Tabriz, Iran

**Keywords:** Apelin, Maternal high-fat diet, Memory, Morris water maze, Troxerutin

## Abstract

**Objective(s):**

Maternal high-fat diet (HFD) is linked with metabolic and cognitive deficits in offspring. Neuroprotective effects of troxerutin, a natural bioflavonoid, have been reported recently. This study aimed to investigate the effects of troxerutin on spatial memory and serum and hippocampal apelin levels in the male offspring of HFD fed mothers.

**Materials and Methods:**

Three-week-old female Wistar rats (n= 40) received HFD or control diet (CD) for 8 weeks. After mating, pregnant animals were divided into two subgroups according to the troxerutin (TRO) supplementation: CD, CD+TRO, HFD, and HFD+TRO. HFD continued to the end of lactation in HFD and HFD+TRO groups. TRO was gavaged (150 mg/kg/day) during pregnancy. After weaning, the male offspring were fed a normal diet until 12 weeks of age. Spatial memory was evaluated in the Morris water maze (MWM) on postnatal day (PND) 90. Total apelin concentration was measured in the serum of maternal rats before mating and after lactation and also in the serum and hippocampus of their male offspring.

**Results:**

Both traveled distance (*P*<0.05) and time spent (*P*<0.05) in the target quadrant were significantly decreased in the offspring of HFD-fed dams, which were reversed by TRO treatment. Moreover, TRO significantly (*P*<0.05) decreased serum apelin levels in dams. Furthermore, TRO treatment in dams significantly (*P*<0.05) increased serum and hippocampal levels of apelin in their offspring.

**Conclusion:**

These results indicated that TRO treatment during pregnancy improved maternal HFD-induced spatial memory impairments of the offspring possibly through modulation of serum and hippocampal apelin levels.

## Introduction

Maternal nutrition during pregnancy and lactation influence the health of mother and child (1). Maternal high-fat diet (HFD) is linked to the high risk of developing metabolic disorders including chronic liver disease, diabetes, high blood pressure, and obesity in offspring (2, 3). Maternal diet can also alter offspring behaviors and cognitive abilities by modifying the intrauterine environment and maternal behaviors (4, 5).

Offspring of obese mothers consuming HFD are vulnerable to mental and behavioral disorders including depression, anxiety, attention deficit hyperactivity disorder, and autism (5, 6). A possible mechanism of increased risk for behavioral disorders is alterations in neural pathways development involved in behavioral regulation (3). It has also been reported that intake of HFD during pregnancy is associated with learning impairments and memory loss in the adult offspring (6, 7). 

Exact mechanisms explaining the link between HFD during pregnancy and memory disorders in infants are not known. Recent findings indicate factors such as the type of nutrient intake (sugar and fat), hormones (insulin and leptin), and inflammatory cytokines affect fetal brain development (3). Development of neurotransmitter systems is also influenced by the levels of circulating cytokines (8, 9). Cytokines also have direct detrimental effects on hippocampal circuitry and cognition (10). 

Apelin, as an adipokine, is secreted by adipocytes. It was initially isolated in 1998 from bovine stomach and defined as an endogenous ligand for an orphan G-protein coupled receptor, APJ (11). Apelin and its receptors are broadly distributed in the peripheral tissues and central nervous system (CNS) particularly in the hippocampus and amygdala, brain regions involved in learning and memory, and hypothalamus (11-13). Expression of apelin is influenced by nutritional status and decreased by fasting and recovered by refeeding (14). Apelin is considered as an anti-obesity peptide which increases the sensitivity of various tissues especially skeletal muscles to insulin, enhances energy consumption, and reduces body fat mass (15, 16). Moreover, apelin has important roles in the regulation of immune response (17), hemodynamic and body fluid homeostasis, angiogenesis, oxidative stress-linked atherosclerosis, and brain signaling (18). It is also reported that apelin is involved in the regulation of memory formation (19, 20), and its active form protects the nervous system from different diseases such as Parkinson’s disease, Alzheimer’s disease, and Huntington’s disease (21).

Troxerutin, a natural bioflavonoid rutin, is present in tea, coffee, cereal grains, and different types of fruits and vegetables (22). Previous studies revealed that it has multiple biological properties including anti-neoplastic, antioxidant, and anti-inflammatory effects (22-25). Our previous studies demonstrated that troxerutin reduces oxidative stress markers, improves spatial learning and memory, and ameliorates synaptic plasticity of dentate gyrus neurons in an amyloid-beta induced rat model of Alzheimer’s disease (26-28). 

To better understand the impact of maternal HFD on offspring behavior, the effect of troxerutin treatment on HFD-induced spatial learning and memory impairments, as well as serum and hippocampal apelin levels in offspring was evaluated.

## Materials and Metods

***Animals and treatments***

The present study was carried out at Tabriz University of Medical Sciences, Tabriz, Iran from March 2016 to Jun 2017. All procedures and experiments were carried out in accordance with the regulations of Tabriz University of Medical Sciences Ethical Committee for the Protection of Animals in Research. Forty virgin female Wistar rats (3 weeks old) were obtained from the colony of Tabriz University of Medical Sciences. Animals were kept in standard conditions, three per cage in a *temperature *(22 ^°^C–25 ^°^C) and humidity (40–60%) controlled room with a 12 hr light/12 hr dark cycle. Food and water were provided *ad libitum* except during the behavioral tests.

Animals were randomly divided into two groups: HFD group, which was fed a high- fat diet (52.0% lipids, 27.1% protein, 20.9% carbohydrate; 4.07 kcal/g) and CD group, which was fed a standard rat chow diet (14.7% lipids, 33.0% protein: 52.2 % carbohydrate; 2.75 Kcal/g) purchased from Behparvar CO, Tehran, Iran. Both groups were fed for 8 weeks before mating. All twenty female animals in each group were housed with adult males overnight for mating, and pregnancy was verified by examining vaginal smears for the presence of vaginal plugs. 

**Figure 1:**
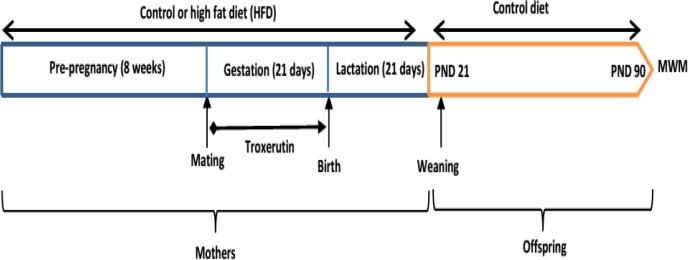
The protocol of the study. PND: postnatal day; MVM: Morris water maze

Then pregnant animals of each group were divided into two subgroups as follows: CD, CD+TRO, HFD, and HFD+TRO**.** Animals in the CD and HFD groups received saline as the vehicle of troxerutin; however, rats in the CD+TRO and HFD+TRO groups were treated with troxerutin (150 mg/kg/day, oral gavage) (29) during the pregnancy. HFD continued to the end of lactation in the HFD and HFD+TRO groups. Troxerutin (TRO) was acquired from Merck (Germany) and all other chemicals and reagents were obtained from commercial sources of the highest quality available. At the end of lactation, male offspring were separated from their mothers and kept in separate cages (4 per cage) in their respective groups. All offspring were fed a normal diet until postnatal day (PND) 90. The protocol of the study is shown in Figure 1.

**Figure 2:**
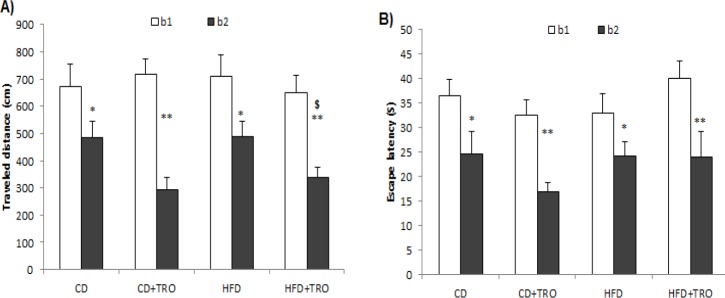
The effect of maternal high-fat diet (HFD) and troxerutin (TRO) treatment on traveled distance (A) and escape latency (B) to find the hidden platform in the offspring groups. Each block represents the average of four consecutive trials. Paired sample t-test; *P<0.05 and **P<0.01 indicate the difference between block 1 (b1) and block 2 (b2) in each group. $ *P*<0.05 indicates the difference between HFD+TRO and HFD groups in block 2. Data are expressed as mean±SEM (n=7). [CD: control diet; TRO: troxerutin; HFD: high fat diet]

**Figure 3:**
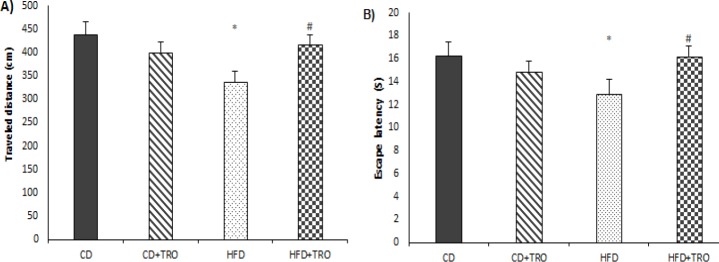
The effect of maternal HFD and troxerutin on the traveled distance (A) and time spent in the target quadrant (B) of the offspring groups in the probe test. Data are expressed as mean±SEM (n=7). One-way ANOVA followed by LSD post hoc test; *P<0.05 vs. CD group, #*P*<0.05 vs. HFD group. [CD: control diet; TRO: troxerutin; HFD: high fat diet]

**Figure 4:**
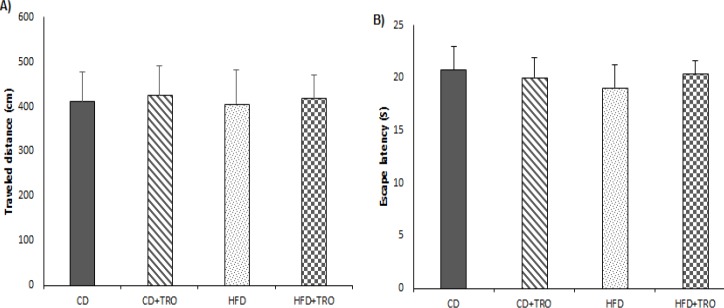
The effect of maternal HFD and troxerutin on the traveled distance (A) and time spent in the target quadrant (B) in the visible platform test in the offspring groups. Data are expressed as mean ± SEM (n=7). There were no significant differences among the four groups. [CD: control diet; TRO: troxerutin; HFD: high fat diet]

**Figure 5:**
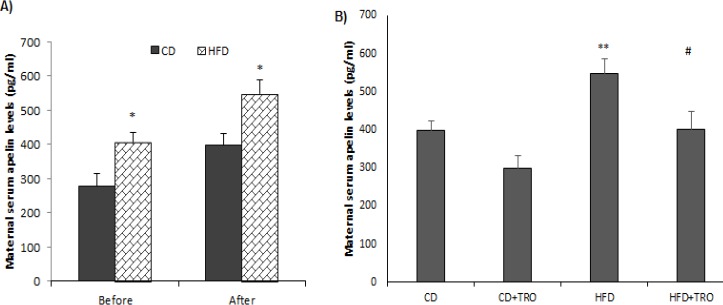
(A) Total serum maternal apelin levels before and after pregnancy. Paired sample t-test; *P<0.05 indicates the difference in the CD groups and HFD groups before and after pregnancy. (B) The effect of HFD and troxerutin on serum total apelin levels of dams at the end of lactation. One-way ANOVA followed by LSD post hoc test; **P0.01 vs. CD group; #*P*<0.05 vs. HFD group. Data are expressed as mean±SEM (n=7). [CD: control diet; TRO: troxerutin; HFD: high fat diet]

**Figure 6:**
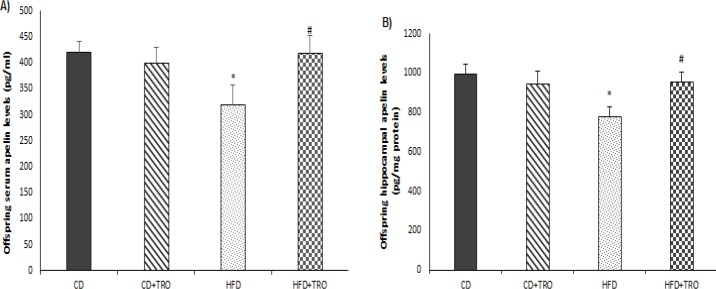
The effect of maternal HFD and troxerutin on serum (A) and hippocampal (B) total apelin levels of offspring groups (PND=90). One-way ANOVA followed by LSD post hoc test; *P<0.05 vs. CD group, #*P*<0.05 vs. HFD group. Data are expressed as mean±SEM (n=7). [CD: control diet; TRO: troxerutin; HFD: high fat diet]

***Morris water maze test***

Animals were transferred to the behavioral test room 30 min before the test. Spatial memory was tested on PND 90 between 10 a.m. and 2 p.m using the MWM apparatus, which was a black circular pool with a diameter of 130 cm and height of 80 cm, filled with water (23 ± 2 ºC) to a depth of 60 cm. The maze was divided into four equal quadrants, and the release points in the quadrants were designated as N, E, S, and W. A hidden Plexiglas platform (10 × 10 cm) was placed in the center of the NW quadrant submerged 1.5 cm below the water surface. Fixed positioned distinctive visual cues were placed at several locations around the maze for facilitating spatial orientation of the animal and kept unchanged throughout the experiments.

On the first day, the training session was performed in two subsequent blocks (four trials in each block and 5 min intervals between the blocks) with four different starting points. The task required animals to swim to the hidden platform guided by the visual cues. The time taken to reach the platform and the traveled distance were measured. Twenty four hours after the last session, rats were subjected to a probe trial which consisted of a 60 sec free-swimming period without a platform. The traveled distance and time spent by animals in search of the platform in the target quadrant were measured. In order to assess the effect of animals’ sensory and motor coordination or the animals’ motivation, the capability of rats to escape to a visible platform was tested 5 min after probe tests (30).

***ELISA assays***

At the end of behavioral testing, animals were deeply anesthetized by intraperitoneal injection of ketamine (60 mg/kg) and xylazine (5 mg/kg). Blood samples were collected from the heart and then centrifuged at 4000 rpm for 10 min. Serum samples were separated and kept at -80 ^°^C until analysis. After decapitation, the brain was rapidly excised and the hippocampus immediately dissected on an ice-cold dissection board. Isolated hippocampal tissues were kept at -80 ^°^C for later measurements. In order to measure total apelin levels, hippocampal samples were homogenized in 1.15% KCl solution, then homogenates were centrifuged (5000 rpm for 10 min at 4 ^°^C) and the supernatants were removed. The total apelin levels in the serum and hippocampal samples were determined using rat-specific apelin (nonspecific sensitivity for other factors) enzyme-linked immunosorbent assay (ELISA) kit (EASTBIOPHARM, China) in accordance with the manufacturer’s assay protocol.

***Statistical analysis***

All results are expressed as mean±SEM for seven animals and analyses were performed using SPSS statistical software version 16. The paired Student’s *t*-test was used in each group for comparison between blocks 1 and 2, and comparing total serum apelin levels of animals before pregnancy and after lactation in mothers groups. For other comparisons in the offspring groups, *one-way ANOVA followed* by *LSD post hoc test was *used. In all cases, *P*-values less than 0.05 were considered statistically significant.

## Results

***Effects of HFD and troxerutin on cognitive performance of offspring in the Morris water maze***

Our results showed that during the training sessions, the performance of all groups improved as indicated by shorter traveled distance and escape latency. The results of paired *t*-test for traveled distance revealed significant differences between two blocks (t= 8.24, df= 27) in the offspring of CD (*P*<0.05), CD+TRO (*P*<0.01), HFD (*P*<0.05), and HFD+TRO (*P*<0.01) groups (Figure 2A). Moreover, the results of escape latency showed significant differences between two blocks (t= 7.61, df= 27) in the offspring of CD (*P*<0.05), CD+ TRO (*P*<0.01), HFD (*P*<0.05), and HFD+TRO (*P*<0.01) groups (Figure 2B). 

Furthermore, the results of one-way ANOVA in block 2 showed that TRO treatment during gestation significantly (*P*<0.05) decreased traveled distance in the HFD+TRO group as compared to the HFD group.

One-way analysis of ANOVA in probe test showed that traveled distance (F _(3, 24) _= 2.98, *P*<0.05) and time spent in target quadrant (F _(3, 24) _= 3.92, *P*<0.05) to find the hidden platform (Figures 3 A and B, respectively) in the HFD offspring were significantly lower than the CD group, indicating memory impairment in this group. However, daily treatments with TRO significantly (*P*<0.05) increased traveled distance and time spent in the target quadrant in the HFD+TRO group compared to the HFD received group, indicating memory improvement in this group.

Furthermore, there were no significant differences in the traveled distance and escape latency to find the visible platform among all the offspring groups. These results suggest that HFD had no effect on sensorimotor and motivational aspects, indicating impairment in spatial memory is due to cognitive deficits (Figures 4A and B). 

***Effects of HFD and troxerutin on maternal total serum apelin levels***

To determine the effects of HFD on maternal endogenous apelin levels, serum concentration of apelin was determined before pregnancy and after pregnancy (Figure 5A), and at the end of lactation (Figure 5B). Our findings demonstrated that serum levels of total apelin in both CD and HFD dams after pregnancy were significantly (*P*<0.05) higher than before pregnancy. Moreover, one-way ANOVA analysis showed that serum apelin levels in the CD group were significantly (*P*<0.05) lower than in the HFD group at the end of lactation. Daily TRO administration (150 mg/kg) during pregnancy could significantly decrease total serum apelin levels in the CD+TRO (*P*<0.05) and HFD+TRO (*P*<0.05) groups in comparison with CD and HFD groups, respectively (Figure 5B).

***Effects of HFD and troxerutin on serum and hippocampal total apelin levels of the offspring***

According to data analysis, HFD offspring groups showed significantly lower serum (F _(3, 24) _=2.25, *P*<0.05) and hippocampal (F _(3, 24) _= 2.44, *P*<0.05) total apelin levels in comparison with the corresponding CD offspring group (Figures 6A and B). However, daily TRO treatments significantly (*P*<0.05) increased serum and hippocampal total apelin levels in the HFD+TRO offspring group as compared to the HFD group.

## Discussion

In the present study, it was demonstrated that maternal HFD prior to breeding and throughout gestation and lactation could have deleterious effects on spatial memory of the offspring. Moreover, HFD decreased serum and hippocampal apelin levels in the offspring. However, chronic treatment with TRO during pregnancy and lactation improved spatial memory and increased apelin levels in the offspring of HFD fed mothers.

Previous studies indicated that HFD prior to breeding in female rats led to obesity and hyperlipidemia in newborn pups, which was associated with oxidative stress and reduced hippocampal neurogenesis (31, 32). Moreover, HFD stimulates stress signaling cascades which can damage the neurodevelopment of offspring (33). Several experimental studies have also demonstrated that chronic HFD is associated with cognitive decline (32, 34). White *et al.* have shown that maternal and offspring HFD consumption increases IL-6, a pro-inflammatory cytokine, in the cortex (32). In the present study, maternal HFD impaired the spatial memory of offspring in the MWM task. Similarly, several studies reported impaired spatial memory in the male offspring of obese mothers in the Barnes maze and MWM tests (4, 32, 35, 36). In another study, young adult male offspring exposed to maternal HFD showed a decreased exploration in the novel object recognition test (37). In contrast to our results, Bilbo and Tsang (38) showed that adult male and female rats born to dams fed HFD performed better on the MWM test compared to the control animals. Different impacts of HFD on memory might be due to different experimental animals or HFD models, age of animals at assessment, and cognitive test paradigms. 

In the present study, oral administration of TRO (150 mg/kg) for 21 days in the HFD pregnant animals effectively protected their offspring against HFD-induced impairment of learning and memory. This finding is consistent with recent studies which demonstrated that TRO attenuates cognitive impairment and oxidative stress induced by administration of amyloid-β or D-galactose in the hippocampus of rats treated with high cholesterol diet (25, 27, 39)**. **Moreover, a recent study has reported that TRO promotes learning and memory in streptozotocin-induced type 1 diabetes by inhibiting oxidative stress response (40). 

Our results also revealed that HFD fed mothers exhibited high levels of apelin in the serum after pregnancy and at the end of lactation as compared to the CD group. It has been demonstrated that nutritional status and insulin-dependent mechanisms of apelin secretion may also have effects on apelin concentration (14). Exposure to high levels of fatty acids can reduce the sensitivity of cells to insulin and induce hyperinsulinemia, which in turn increases apelin secretion from the adipose tissue (41). Zhang *et al.* also demonstrated that HFD feeding increased fasting blood glucose and serum insulin levels in mice (42). Therefore, increased serum apelin levels in the HFD dams could be explained by this mechanism. On the other hand, we found that administration of TRO during gestation decreased apelin levels in the mother’s serum. In support of our finding, evidence shows that TRO has a restoring effect on adipokine protein expression in the liver of HFD-treated rats (43). Recently, a study also reported that TRO inhibits inflammatory cytokines release and declines adiponectin levels in the HFD-received mice. Furthermore, TRO treatment reduces serum insulin levels in the HFD-treated mice (42). It has also been reported that TRO can prevent obesity by improving insulin signaling pathway, and it returns blood glucose, fatty acids, and cholesterol levels to the normal levels (44). Therefore, it is likely that TRO through these mechanisms reduced maternal apelin levels.

Here, we also showed that offspring’s apelin levels in the HFD group decreased as a result of maternal HFD consumption which was increased in the HFD+TRO group. Researchers (45) reported that in utero HFD exposure changes adipocytokine promoter epigenetic and leads to abnormal adipocytokine levels in the offspring. Therefore, it is likely that maternal HFD modifies post-translational processes and cellular mechanisms involved in the apelin release. Moreover, maternal HFD augments hypothalamus-pituitary axis (HPA) activity and increases corticosterone levels in their offspring (46). Previous studies also showed that glucocorticoids suppress apelin gene expression in adipocytes in a dose-dependent manner which reduces apelin production (47, 48). Therefore, it seems that high cortisol levels of the HFD-fed offspring group have suppressed the apelin production in the HFD offspring, and TRO has increased apelin levels in the offspring through suppression of cortisol.

Apelin and its receptors are widely distributed in the brain regions involved in learning and memory functions, demonstrating that apelin may participate in the regulation of memory processes (12, 13, 49). In this study, low serum and hippocampal apelin levels were allied with spatial memory impairment in the HFD offspring suggesting that low apelin levels are probable mediators of HFD-induced cognitive disruption. A study (20) showed that intracerebroventricular (ICV) administration of apelin-13 blocks short-term memory formation and long-term memory consolidation in the novel object recognition task. Nevertheless, researchers (50) reported that administration of apelin-13 (2 μg) facilitates learning and memory consolidation in the passive avoidance learning paradigm. Likewise, a recent study (51) reported that central injection of apelin improved memory in the novel object recognition test in the stressed rats. 

Furthermore, elevation of the serum and hippocampal apelin levels was accompanied with improved memory of the HFD+TRO offspring group. Anti-inflammatory and antioxidant properties of TRO have also been proven in several studies (22-24). Therefore, it seems that chronic TRO treatment improves spatial memory through increasing the apelin levels and decreasing the pro-inflammatory cytokines in the hippocampus**. **

## Conclusion

TRO reversed cognitive deficits in the adult male offspring of HFD fed mothers. This effect may be partly mediated by modification of apelin levels. Further studies are needed to elucidate the exact mechanism of TRO action in this regard.
